# Thymoquinone Alleviates the Experimental Diabetic Peripheral Neuropathy by Modulation of Inflammation

**DOI:** 10.1038/srep31656

**Published:** 2016-08-22

**Authors:** Long Chen, Bing Li, Biqin Chen, Yiye Shao, Qiong Luo, Xiaohong Shi, Yinghui Chen

**Affiliations:** 1 Department of Neurology, Jinshan Hospital, Fudan University, Shanghai 201508, P. R. China; 2 Department of Neurology, Shanghai Medical College, Fudan University, Shanghai 200032, P. R. China; 3 Center Laboratory, Jinshan Hospital, Fudan University, Shanghai 201508, P. R.China; 4 Department of Pediatric, Jinshan Hospital, Fudan University, Shanghai 201508, P. R.China; 5 Department of Endocrinology, Jinshan Hospital, Fudan University, Shanghai 201508, P. R.China

## Abstract

Thymoquinone has been reported to exhibit antioxidant and anti-inflammatory effects. Inflammation plays an important role in pathogenesis of diabetic peripheral neuropathy. This study investigated the effects of TQ on proliferation and apoptosis of Schwann cells exposed to high glucose conditions and electrophysiological and morphological changes of the sciatic nerve in a DPN rat model as well as relevant inflammatory mechanism. Cell proliferation and apoptosis of Schwann cells were measured using the Cell Counting Kit-8 and flow cytometry. DPN model was established in streptozotocin-induced diabetic rats. Nerve conduction velocity was measured before and after treatment. Morphologic changes were observed by H&E staining and transmission electron microscopy. COX-2, IL-1β, IL-6, and Caspase-3 expression was investigated by western blotting and Bio-Plex Pro^TM^ Assays. Finally, TQ alleviated the inhibition of Schwann cell proliferation and protected against Schwann cell apoptosis. It improved nerve conduction velocity, and alleviated the DPN-induced morphological changes and demyelination of the sciatic nerve. COX-2, IL-1β, IL-6 and Caspase-3 expression in sciatic nerve or isolated cultured Schwann cells, were also decreased by TQ. These results indicate TQ has a protective effect on peripheral nerves in a DPN rat model. The mechanism may be mediated partly by the modulation of the inflammatory reaction.

Diabetes mellitus is one of the most common metabolic disorders. Type 2 diabetes mellitus (T2DM) accounts for more than 90% of cases of diabetes. According to the International Diabetes Federation (IDF) atlas 2013, there are 381.8 million diabetic patients aged 20–79 years, and this is projected to increase by 55% to 591.9 million by 2035^1^. Diabetic peripheral neuropathy (DPN) is one of the most common chronic complications of diabetes mellitus and often manifests as a distal, symmetric, sensorimotor neuropathy^2^. DPN mainly causes sensory, motor, and autonomic dysfunction^3^, and can lead to ulcers, limb amputation, and death^4^,^5^. However, the pathogenesis of DPN has not been clarified. It is accepted that multiple factors related to chronic uncontrolled hyperglycemia contribute to the development of DPN, including the polyol pathway, advanced glycation end-product formation, oxidative stress, and activation of protein kinase C, and so on^6^,^7^. Recent evidence demonstrates that inflammation is closely related to diabetes and its complications^8^,^9^. Our previous study found elevated expression of the tumor necrosis factor alpha (TNF-α) cytokine in the serum of DPN model rats, and revealed that a TNF-α inhibitor relieved the symptoms in DPN rats^10^. AS main proinflammatory cytokines in inflammatory reaction, IL-6 and IL-1beta are involved in development of many diseases. The elevated levels of IL-6 and IL-1β are found in diabetic rat sciatic nerve suggesting that inflammation is involved in the occurrence of DPN^11^. Therefore, moderate anti-inflammatory therapy may have an effect on DPN. However, nonsteroidal anti-inflammatory drugs (NSAIDs) have limited clinical application due to toxic side effects^12^. Thus, it is extremely important to explore new, safe, and effective anti-inflammatory therapeutic drugs.

Thymoquinone (TQ) is a bioactive monomer derived from black seed (Nigella sativa) oil. TQ has been reported to exhibit many pharmacological effects, including immunomodulatory, anticancer, antioxidant, and anti-inflammatory effects^13^,^14^,^15^,^16^. Previous studies show that TQ suppresses the inflammatory reaction and oxidative stress to relieve injury in pancreatic tissue and diabetic nephropathy in streptozotocin-induced diabetic rats^17^,^18^. Kanter, M had proved that thymoquinone had beneficial effects on histopathological changes of sciatic nerves in STZ-induced diabetic peripheral neuropathy rats by regulating oxidative stress^19^. But, it is not clear whether inflammatory response was also involved in this process, after all, thymoquinone has a strong anti-inflammatory effect while inflammatory reaction participates in the pathogenesis of DPN. Thus, we speculated that TQ could alleviate peripheral nerve injury in a rat model of DPN from the point of anti-inflammation.

Here, we report that TQ protects against glucose-induced apoptosis of Schwann cells and inhibition of Schwann cell proliferation in cultured cells. It also improves nerve conduction velocity, and alleviates pathological morphology changes and sciatic nerve demyelination in DPN rats. Differential expression of COX-2, IL-1β, IL-6, and Caspase-3 between TQ-treated and untreated rats suggests that these effects may be mediated by the inflammatory response.

## Results

### TQ alleviated the inhibition of Schwann cell proliferation following exposure to a high of concentration glucose

**Figure 1:**
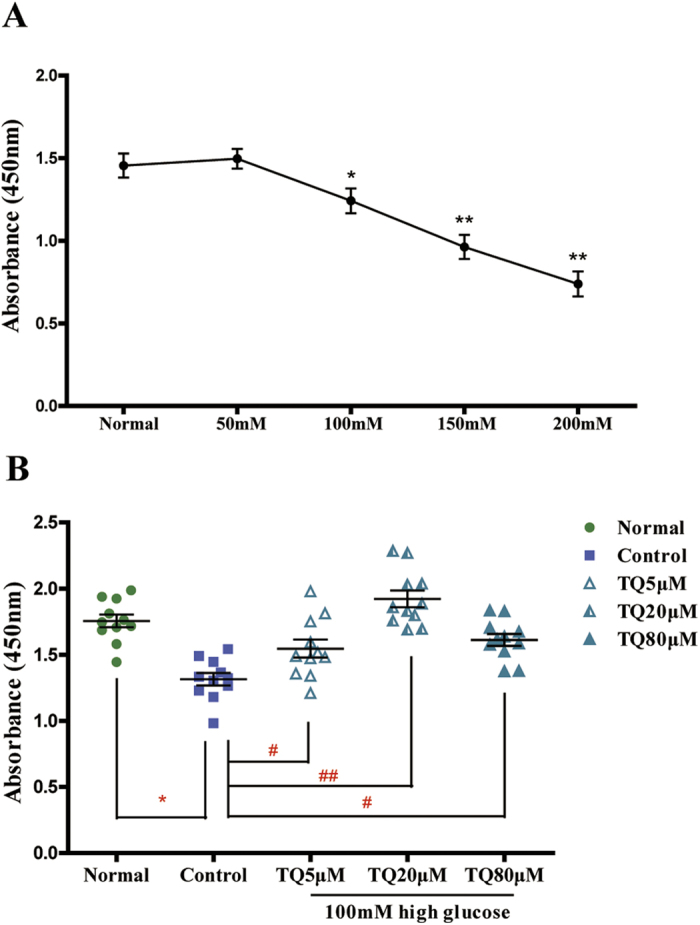
Effect of thymoquinone (TQ) on Schwann cell proliferation induced by high glucose. (**A**) RSC96 cells were treated with 25 (the normal group), 50, 100, 150, or 200 mM high glucose for 24 h. *p *<* 0.05, **p *<* *0.01* versus normal group. (**B**) RSC96 cells were induced by 100 mM high glucose and treated with 0, 5, 20, or 80 μM TQ for 24 h. Cells in the normal group were treated with 25mM glucose for 24 h. *p *<* *0.05* versus the normal group, ^*#*^*p* *<* *0.05* versus control group, ^*##*^*p* *<* *0.01* versus control group.

In the initial stage of the experiment, CCK-8 was performed to measure cell proliferation under different concentrations: 25 (normal group), 50, 100, 150, and 200mM glucose treatment. Compared with the normal group (25 mM), the 50 mM concentration of glucose resulted in no obvious difference in cell proliferation, though there was a trend toward promoting proliferation that was not statistically significant (*p* > 0.05). With the increase of glucose concentration, cell proliferation showed a concentration-dependent inhibition. Treatment with 100, 150, and 200 mM glucose significantly decreased cell viability compared with the normal group (*p* *<* 0.05; Fig. 1A). However, when treated with different concentrations of TQ, the glucose-induced inhibition of Schwann cell proliferation was alleviated (Fig. 1B). Compared with control group (0μM TQ), cell cultures exposed to TQ at 5, 20, and 80 μM for 24 h under 100 mM glucose condition showed 18%, 46.5% and 23% increases in cell proliferation, respectively. The effect of TQ was not dose-dependent. TQ at concentrations of 5 and 80 μM showed a mild effect on cell proliferation (*p* *<* 0.05), but the effect of 20 μM TQ was highly significant (p *<* 0.01).

### TQ protected against high glucose-induced Schwann cells apoptosis

**Figure 2:**
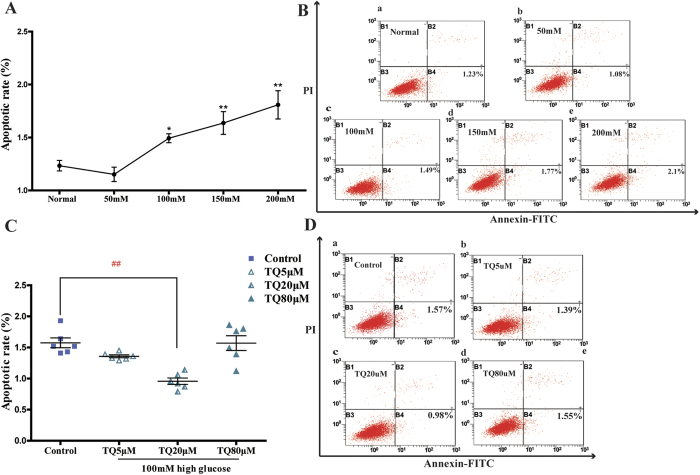
Thymoquinone (TQ) protected against high glucose-induced Schwann cell apoptosis. (**A,B**) RSC96 cells were treated with 25 (the normal group), 50, 100, 150, or 200 mM high glucose for 24 h. (**A**) Apoptotic rate of Schwann cells induced by high glucose. (**B**) Percentages of apoptotic Schwann cell analyzed by flow cytometry. a, b, c, d, e respectively represent normal, 50, 100, 150, 200mM high glucose. *p < 0.05, **p < *0.01* versus normal group. (**C,D**) RSC96 cells were treated with 100mM of high glucose and 5, 20, or 80 μM TQ for 24 h. (**C**) Apoptotic rate of high glucose-induced Schwann cells treated with TQ. (**D**) Percentages of apoptotic Schwann cells with TQ treatment analyzed by flow cytometry. a, b, c, d represent control (100mM) high glucose and 5, 20, and 80 μM TQ respectively. ^*##*^*p* < *0.01* versus the control group.

As shown in Fig. 2, the apoptosis rate of Schwann cells exhibited a downward trend following treatment with 50 mM high glucose compared with the normal glucose concentration but there was no statistical significance (*p* > 0.05). With the increase of glucose concentration, the apoptosis rate elevated significantly. The percentage of Schwann cells undergoing apoptosis under the conditions of 100, 150, and 200 mM glucose were 1.50% ± 0.13%, 1.64% ± 0.26%, and 1.81% ± 0.33%, respectively, which were 21%, 32%, and 46% higher than in the control group, respectively (Fig. 2A,B). When cells were treated with TQ at 5, 20, and 80 μM for 24 h in the 100 mM high glucose condition, the apoptosis rate significantly decreased in the TQ 20 μM group compared with the other groups (*p* *<* 0.01) (Fig. 2C,D). There were no obvious differences between the group treated with the lowest concentration of TQ (5 μM) and the control group (100 mM glucose; *p* > 0.05). Likewise, the highest concentration of TQ (80 μM) showed no protective effect (*p* > 0.05).

### TQ decreased the expression of COX-2 induced by high glucose and lipopolysaccharide (LPS)

**Figure 3:**
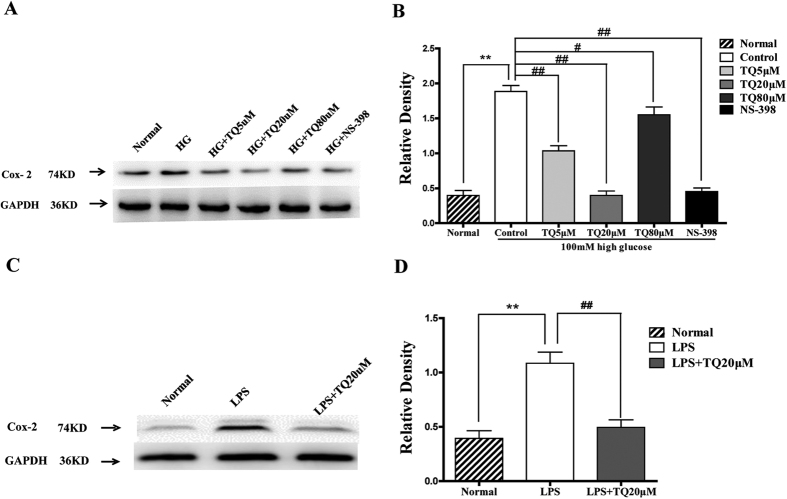
Thymoquinone (TQ) decreased the expression of COX-2 induced by high glucose and lipopolysaccharide (LPS). (**A,B**) RSC96 cells were induced by 100mM glucose for 24 h, then cells were treated with 5, 20, or 80 μM TQ and 10 μM NS-398 for 24 h. (**A**) A representative western blot of COX-2 is shown. HG: high glucose. (**B**) The relative density of COX-2 expression. **p < *0.01* versus the normal group; ^*#*^*p* < *0.05* versus the control group, ^*##*^*p* < *0.01* versus the control group. (**C,D**) RSC96 cells were induced by 1 μg/L LPS and treated with 20 μM TQ for 24 h. (**C**) A representative western blot of COX-2 is shown. (**B**) The relative density of COX-2 expression. **p < *0.01* versus the normal group; ^##^*p* < *0.01* versus the control group.

To verify whether TQ could inhibit the inflammatory reactions induced by high a concentration of glucose and LPS, we examined the levels of COX-2, which plays an important role in inflammatory response in Schwann cells treated with 100 mM glucose and 1 μg/mL LPS. Our results showed that COX-2 levels were low in normal glucose, but when Schwann cells were exposed to high glucose, the COX-2 level was significantly up-regulated (*p* *<* 0.01). TQ at concentrations of 5 and 20 μM significantly reduced the expression of COX-2 (*p* *<* 0.01). Of note, at the 20 μM concentration, TQ downregulated the expression of COX-2 induced by high glucose as effectively as the selective COX-2 inhibitor NS-398 (Fig. 3A,B). The same results were found when Schwann cells were treated with 1 μg/ml LPS and 20 μM TQ. LPS significantly upregulated the expression of COX-2 compared with the normal group (*p* *<* 0.01). After the administration of TQ, the expression of COX-2 induced by LPS was significantly inhibited (*p* *<* 0.01) (Fig. 3C,D).

### Validation of the diabetic peripheral neuropathy rat model

In our study, the DPN model was established by a high-fat, high-sugar diet for 6 weeks, followed by a single dose of STZ intraperitoneal injection. After 72 h of STZ injection, T2DM rats were validated by their higher blood glucose levels compared with normal animals. DPN group rats had high baseline fasting blood glucose levels compared with the normal group rats (*p* *<* 0.05) and there was no significant difference between the treated DPN groups (*p* > 0.05). After treatment for 6 weeks, blood glucose levels of the TQ treated groups were decreased, especially in the DPN+TQ5 group, but no significant differences in fasting blood glucose levels were found (p > 0.05). And there was no significant difference in the levels of blood glucose before (0 week) and after (6 weeks) the injection of VitB_12_ (*p* > 0.05) (Table 1).

### TQ rescued the decrease of motor nerve conduction velocity and sensory nerve conduction velocity in DPN rats

**Figure 4:**
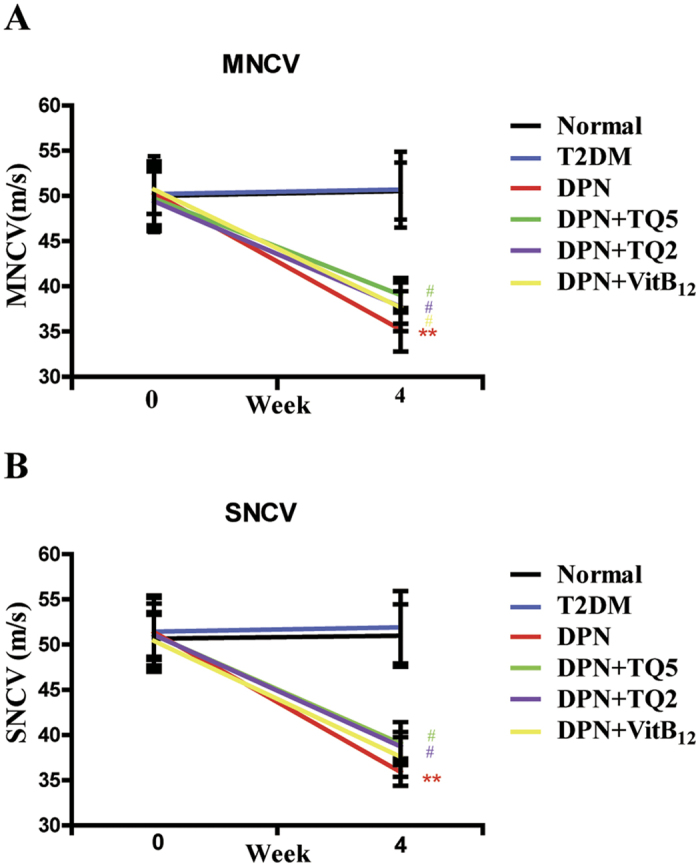
Thymoquinone (TQ) rescued the decrease of motor nerve conduction velocity and sensory nerve conduction velocity in DPN rats. (**A**) Changes of motor nerve conduction velocity among groups at 0th week and 6th week. (**B**) Changes of sensory nerve conduction velocity among groups at 0th week and 6th week. **p < 0.01 versus normal group; ^#^*p* < 0.05 versus the diabetic peripheral neuropathy (DPN) group.

In order to estimate the effect of TQ on nerve electrophysiology, NCV was examined. As shown in Fig. 4, there were no statistically significant differences in either motor NCV (MNCV) or sensory NCV (SNCV) among any groups before treatment (*p* > 0.05). As the extension of time, MNCV and SNCV gradually decreased in the DPN group (*p* *<* 0.05); both motor and sensory NCV in the DPN+TQ2 and the DPN+TQ5 group were significantly higher than that in the DPN group after treatment at sixth weeks (*p* *<* 0.05). Further, the MNCV in the VitB_12_ group was higher than that in the DPN group after treatment (*p* *<* 0.05). Though the SNCV in the VitB_12_ group was also higher, this effect was not statistically significant (*p* > 0.05). (Fig. 4A,B).

### TQ attenuated the morphological changes in the sciatic nerve in DPN rats

**Figure 5:**
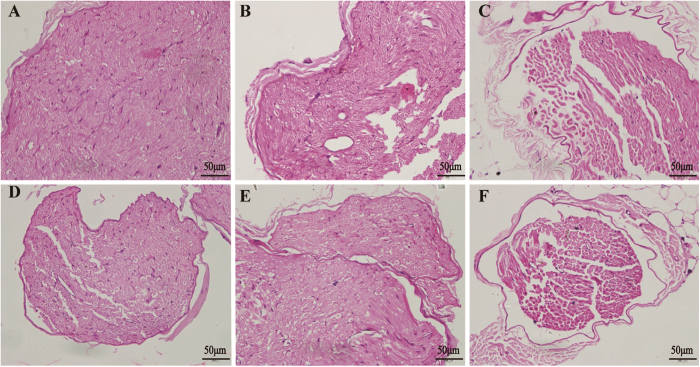
Hematoxylin and eosin staining revealed that thymoquinone (TQ) attenuated diabetic peripheral neuropathy (DPN)-induced pathological changes. (**A**) Normal group; (**B**) T2DM group; (**C**) DPN group; (**D**) DPN+TQ5 group; (**E**) DPN+TQ2 group; (**F**) DPN+VitB12 group. T2DM: type 2 diabetes mellitus; TQ2: 2 mg/kg TQ; TQ5: 5 mg/kg TQ; VitB12: vitamin B12. Panel shows longitudinal sections with 400X amplification (scale bar = 50 μm).

H&E staining was performed to observe the histopathology of the rat sciatic nerve. In the normal group rats, the myelinated nerve fibers were similar in size. Myelin appeared dense, round, and uniform with ordered lamellar structures presenting neither axonal shrinkage nor swelling. In the DPN group rats, the myelin sheath of the myelinated nerve fibers was thin, loose, and disorganized and exhibited vacuolar-like defects. Some nerve fibers in the sciatic nerve appeared demyelinated. Lamellar spaces were expanded and separated from each other and visible signs of axonal atrophy were evident. The endoneurial capillary displayed thick walls and irregular lumen. The histopathological morphology was improved in DPN+TQ2, DPN+TQ5 and DPN+ViB_12_ groups compared with the DPN group. Further, myelinated nerve fiber density was higher, myelin structure was more complete, and vacuolar degeneration was lower, especially in the DPN+TQ5 group (Fig. 5). Though the morphology in the treated group was greatly improved, it was not as good as the normal group yet.

**Figure 6:**
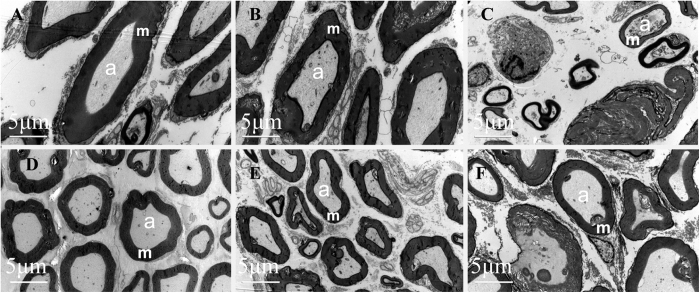
Transmission electron microscopy revealed that thymoquinone (TQ) attenuated diabetic peripheral neuropathy (DPN)-induced pathological changes. (**A**) Normal group; (**B**) T2DMgroup; (**C**) DPN group; (**D**) DPN+TQ5 group; (**E**) DPN+TQ2 group; (**F**) DPN+VitB12 group. T2DM: type 2 diabetes mellitus; TQ2: 2 mg/kg TQ; TQ5: 5 mg/kg TQ; VitB12: vitamin B12; m: myelin, a: axon. Panel shows longitudinal sections with 3000X amplification (scale bar = 5 μm).

Finally, transmission electron microscopy was used to observe the ultrastructure of myelin. In the normal group rats the cross-section of the sciatic nerve had uniform and dense myelination with structural integrity presenting concentric light and dark circles in lamellar structures and axonal shrinkage and swelling. In the DPN group rats the myelin structure was disorganized and axonal shrinkage was present. There was a visible degree of lamellar fracture, acute demyelination, and separation of the myelin sheath. The number and degree of axonal shrinkage and degeneration of myelin of the nerve fibers in the DPN+TQ2, DPN+TQ5, and DPN+ViB_12_ groups was lower and less severe than that in the DPN group (Fig. 6).

### TQ down-regulated expression of COX-2 and Caspase-3 in the DPN rats

**Figure 7:**
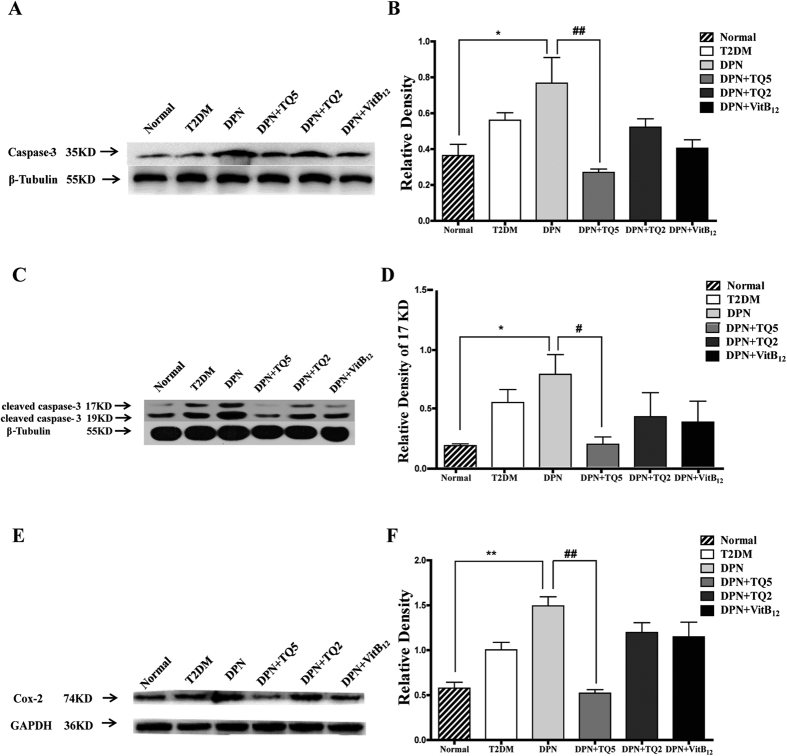
Thymoquinone (TQ) downregulated the expression of COX-2 and Caspase-3 in diabetic peripheral neuropathy (DPN) rats. (**A,B**) Expression of Caspase-3 in rat sciatic nerve. (**A**) Representative western blot of Caspase-3. (**B**) The relative density of Caspase-3 expression. *p < *0.05* versus the normal group; ^*##*^*p* < *0.01* versus the control group. (**C,D**) Expression of cleaved Caspase-3 in rat sciatic nerve. (**C**) Representative western blot of cleaved Caspase-3. (**D**) The relative density of cleaved Caspase-3 of 17KD expression. *p < *0.05* versus the normal group; ^*#*^*p* < *0.05* versus the control group. (**E,F**) Expression of COX-2 in rat sciatic nerve. **(E**) Representative western blot of COX-2. (**F**) The relative density of COX-2 expression. **p < 0.01 versus the normal group; ^#*#*^*p* < 0.01 versus the control group.

Compared with the normal group, COX-2 and Caspase-3 including the cleaved Caspase-3 expression in the T2DM group was higher, but this effect was not statistically significant (*p* > 0.05). However, Caspase-3 and its cleaved product expression in the sciatic nerve of the DPN group showed a significant statistical increase (p < 0.05), while COX-2 expression was more significant (p < 0.01). Compared with the DPN group, the expression of these proteins in the DPN+TQ2 and DPN+VitB_12_ group was lower, but the differences did not reach statistical significance (*p* > 0.05). However, COX-2 and Caspase-3 including its cleaved product expression were significantly decreased in the DPN+TQ5 group (*p* < 0.05; Fig. 7).

### TQ down-regulated expression of IL-6 and IL-1β in the plasma of DPN rats

As shown in Table 2, although they were undetected in the normal group, IL-6 and IL-1β expression in the T2DM and DPN groups was remarkably up-regulated compared with the normal group (*p* *<* 0.05), but there was no significant difference between the T2DMand DPN groups. IL-6 expression was significantly downregulated in the DPN+TQ5, DPN+TQ2, and DPN+VitB_12_ groups compared with the DPN group (*p* *<* 0.05). IL-1β expression was significantly decreased only in the DPN+TQ5 group (*p* *<* 0.05).

## Discussion

DPN is one of the most common chronic complications of T2DM. About 50% of the population with T2DM will experience damage to the peripheral nerves. It has caused a significant economic burden to society^20^. The mechanism of the development of DPN has not yet been fully elucidated. Studies have shown that inflammatory reaction may play a role in the development of DPN^21^. Our previous study also confirmed the point that there is a great relationship between inflammation and DPN. Because of the side effects in the use of anti-inflammatory drugs clinically, to discover new and effective anti-inflammatory drugs for the treatment of DPN is extremely needed.

As stated previously, TQ has been reported to exhibit remarkable antioxidant and anti-inflammatory effects. However, the effect of TQ on DPN has rarely been reported. In order to verify the protective effect of TQ on DPN and the mechanism involved, we conducted the experiment *in vitro* and *in vivo*.

A large number of studies demonstrate that Schwann cells are closely related to the pathogenesis of diabetic peripheral neuropathy^22^,^23^. Schwann cell abnormalities may cause nerve dysfunction, such as reduced nerve conduction velocity, axonal atrophy and impaired axonal regeneration. Based on the key role of Schwann cells in DPN, we chose Schwann cells as the object of our *in vitro* study. We observed that the proliferation of Schwann cells was inhibited by high concentrations of glucose, consistent with other researchers’ reports^24^. However, when treated with TQ, the inhibition of Schwann cell proliferation induced by high glucose was alleviated, especially following treatment with 20 μM TQ. Currently, it remains unclear in pathophysiology that why high glucose could repress Schwann cell proliferation. Studies find that high glucose may cause inhibitory effect on SC proliferation through ERK signaling activation or ERK1/2 MAPK signaling and so on^25^,^26^,^27^. Hence, it is speculated that TQ may promote Schwann cells proliferation by modulating some signaling pathway. Proliferation and migration of Schwann cells is an important physiological process in repairing nerve fiber damage. Studies observed that if proliferation of Schwann cells was inhibited, their migration ability was also weakened^28^. Thus, TQ may play an important role in repairing nerve fibers by promoting proliferation and migration of Schwann cells in DPN.

Further, our study indicated that TQ could not only promote proliferation, but also inhibit the apoptosis of Schwann cells induced by high glucose. When the glucose concentration was greater than 100 mM, the rate of Schwann cell apoptosis gradually increased with the elevated glucose concentration. Though the total apoptosis rate was not high, this may be due to the fact that we cultured the Schwann cells in high glucose for a short time only: 24 h. However, statistically significant differences were observed between the high glucose group and the normal glucose group. After treatment with TQ, the apoptosis rate of Schwann cells induced by high glucose was significantly decreased in the TQ 20 μM group.

However, the highest concentration of TQ (80 μM) showed no protective effect, perhaps because the toxic effect outweighed the treatment effect at this concentration. Apoptosis of Schwann cells plays a major role in demyelination and allergic neuritis in demyelinating diseases^29^,^30^,^31^. By inhibiting apoptosis of Schwann cells, TQ could help to repair peripheral nerves, reduce demyelination of peripheral nerves, and slow down the process of peripheral nerve degeneration.

To study the mechanisms involved in these effects on Schwann cells, we observed the effect of TQ on the inflammatory response. In the present study, we found that TQ decreased the expression of COX-2 in Schwann cells cultured in a high concentration of glucose, which illustrates that TQ could decrease the inflammatory reaction induced by high concentration glucose. To further verify the anti-inflammatory effect of TQ, we observed the effect of TQ on the inflammatory response induced by LPS. Likewise, we found that expression of COX-2 was significantly inhibited. Our data demonstrated that TQ showed a strong anti-inflammatory effect that was as efficient as the selective COX-2 inhibitor NS-398.

Based on previous results, we speculate that TQ could protect peripheral nerves, and promote their repair in DPN. In order to verify this speculation, we observed the protective effect of TQ on DPN *in vivo*. In the DPN rat model, we found a decreased NCV and pathologic morphology changes in the sciatic nerve characterized by demyelination and diffuse shrinkage in myelin nerve fibers compared with the normal group rats. However, after treatment with TQ for 6 weeks, both the NCV and the pathological morphology of the sciatic nerve were significantly improved, especially at a dosage of 5mg/kg. The same effect could also be found in VitB_12_ treated groups. As a kind of classic neuroprotective drug, VitB_12_ is one of the most important mediators of nervous system function. A VitB_12_ deficiency expresses itself by a wide variety of neurological manifestations such as paraesthesias, skin numbness, coordination disorders and reduced nerve conduction velocity^32^. In the clinic, it is often used to accelerate recovery of peripheral nerves^33^. So, we could find the phenomenon that TQ showed an equal effect as Vitamin B_12_ or even better. Besides, there were no significant differences in blood glucose changes between the diabetic rats and the treated DPN groups, although the blood glucose concentration of the TQ treated groups was decreased. This indicates that the protective effect of TQ was not mediated through the regulation of blood glucose. The results of the present study suggested that TQ may be effective in impeding pathogenic processes occurring in the DPN rat induced by STZ injection.

Furthermore, our results showed that IL-1β and IL-6 expression in plasma and COX-2 and Caspase-3 including its activated product expression in the sciatic nerve was also down-regulated in DPN rats by TQ. These results suggest that TQ could reduce the inflammatory reaction and cell apoptosis in the sciatic nerve of DPN rats and have a protective effect on DPN. The elevated levels of COX-2, IL-6, and IL-1β in diabetic rat sciatic nerve suggest that inflammation is involved in the occurrence of DPN^11^. Glucose-mediated alteration of COX-2 pathways plays a critical role in mediating peripheral nerve dysfunction in diabetes^34^. As a key enzyme in inflammatory process, COX-2 overexpression induced by high glucose can activate a series of down-stream inflammatory responses. The subsequent prostaglandin E2 (PGE2) activity could induce pro-apoptotic molecules such as Caspase-3, Bax, and- Caspase-9 to be overexpressed, which can generate a series of activation reactions such as cleaved caspase-3 and finally lead to cell apoptosis^35^. Further, COX-2 activation itself promotes the production of reactive oxygen species and causes oxidative stress in the diabetic peripheral nerves, also inducing cell apoptosis^36^. What is more important is that researchers demonstrated that down-regulating COX-2 expression may be useful for preventing or delaying DPN^37^. IL-6 and IL-1β are important inflammatory cytokines involved in oligodendrocyte toxicity and demyelination in DPN. Particularly, increased IL-6 can lead to endothelial cell dysfunction, which is a key process that leads to the development of atherosclerosis and eventually leads to neurological ischemia and damage^38^. If the inflammatory reaction is inhibited, the symptoms of DPN can be relieved, just as our previous study and others have demonstrated^10^. Thus, TQ may play a protective role in DPN due to its anti -inflammatory effect.

A large number of studies have indicated that oxidative stress is also involved in diabetes and its complications. As we have mentioned above, TQ also has excellent antioxidant ability^39^,^40^,^41^. However, in the current study, we only focused on the anti-inflammatory effects of TQ. Thus, further study is needed to verify the role of TQ in DPN.

## Conclusions

Taken together, our results indicate that TQ has a protective effect on peripheral nerves in the DPN rat. The mechanism may be mediated, in part, by modulation of the inflammatory reaction. The present study may provide experimental evidence to merit further exploration of the possible use of thymoquinone as a therapeutic approach in the treatment of diabetic peripheral neuropathy.

## Materials and Methods

### Cell culture and treatment

RSC96 cells (rat Schwann cells) were obtained from the Cell Resource Center of the Shanghai Institute for Biological Sciences, Chinese Academy of Sciences and cultured in Dulbecco’s modified Eagle’s medium (DMEM, GIBCO, USA) containing 25 mM glucose supplemented with 10% bovine serum (GIBCO-16000–044), in a humidified 5% CO2, 95% air, 37 °C incubator. The effect of a high glucose concentration on Schwann cells was investigated by comparing the following conditions: normal concentration of glucose (25 mM glucose, NG) and high concentrations of glucose (50, 100, 150, and 200 mM glucose, HG). Cells were cultured for 24 h after the addition of glucose. TQ treatment was conducted as follows: 100 mM glucose plus TQ at different concentrations (5, 20, and 80 μM) 4 h after the addition of glucose, for 24 h.

### Cell proliferation assay using the Cell Counting Kit-8 (CCK-8) and determination of cell apoptosis by flow cytometry analysis

Cell proliferation was determined using cell counting kit-8 (Dojindo, Japan) as previously described^42^. For each group, cells were seeded in a 96-well plate at a density of 1 × 10^4^ cells/well and four repeated wells were made. Three experiments were conducted.

RSC96 Schwann cells were seeded in a 6-well plate and treated as previously described. Cell apoptosis was detected by Annexin V, FITC Apoptosis Detection Kit (Dojindo Laboratories, Japan) according to the manufacturer’s instructions. Stained cells were analyzed using a FACScalibur Flow cytometer (Beckman coulter). For all assays, 15,000 cells were counted.

### Experimental animals and grouping

Adult male Wistar rats weighing 160–180g were purchased from the SIPPR-BK Lab Animal Co., Ltd. (Shanghai, China). All animal experiments were approved by the Ethic Committee of Animal Care of Jinshan Hospital. All experimental methods were carried out in accordance with the National Institutes of Health Guide for the Care and Use of Laboratory Animals. These animals were randomly divided into six groups: normal control group (n = 10), T2DM group (n = 10), DPN control group (n = 10), DPN+TQ 2 mg/kg group (n = 10), DPN+TQ 5 mg/kg group (n = 10), and DPN + vitamin B_12_ (VitB_12_) 600 μg/kg group (n = 10).

### Establishment of a DPN model

The rat model of DPN was established as previously described^10^. Briefly, after being fed a high-fat, high-sugar diet (normal diet mixed with 10% lard and 20% sucrose) for 6 weeks, diabetes was induced by a single intraperitoneal administration of STZ (30 mg/kg; Sigma) in 0.1 M citrate buffer (pH 4.2) after overnight fasting. Diabetes was confirmed by measuring blood glucose level (>16.7 mmol/L) 72 h after STZ administration. Once the diabetes was induced, the diabetic animals were divided into DPN group and DPN+TQ or VitB12 groups. The DPN group animals were continuously fed with a high-fat, high-sugar diet for another 6 weeks to generate DPN animals. But the DPN+TQ or VitB12 groups animals were intraperitoneally injected with varying concentrations of TQ (274666, Sigma; 2 mg/kg and 5 mg/kg, dissolved in 10% anhydrous ethanol) or VitB12 (Shanghai No.1 Biochemical & Pharmaceutical Co., Ltd; 600 μg/kg), once every 2 days, except with a high-fat, high-sugar diet for 6 weeks. The DPN model was validated by measuring lowered nerve conduction velocity (NCV, *<* 40 m/s). The normal control group rats were given the normal diet all the time in the experiment. The T2DM group rats were firstly given the normal diet for 6 weeks, after that, they were fed a high-fat, high-sugar diet for 6 weeks. Three days before scarification, the rats were given intraperitoneal administration of STZ of 30 mg/kg and blood glucose level was measured 72 h after STZ administration. At the end of the experiment, the animals were sacrificed under chloral hydrate anesthesia and sciatic nerves were obtained. In addition, we monitored blood glucose levels during the experiment.

### Measurement of nerve conduction velocity

NCV was measured in the sciatic nerve. At the end of the sixth week, all animals were anesthetized with 10% chloral hydrate (0.3 ml/100 g) and fixed in the prone position. Motor nerve conduction velocity (MNCV) and sensory nerve conduction velocity (SNCV) were recorded as previously reported^10^. The NCV of each group was measured three times to calculate the mean value.

### Morphological observations

#### Preparation of sciatic nerve tissue, staining, and transmission electron microscopy

Isolated bilateral sciatic nerves were fixed with 4% formaldehyde and embedded in paraffin. Sciatic nerve samples were sectioned (5 μm thick) with a rotary slicer (LEICA RM2135, Wetzlar, Germany). Hematoxylin and eosin stain (H&E) was used to evaluate neuronal damage and myelination status. Transmission electron microscopy observation was carried out as previously reported^10^.

### Western blot

Total protein extracted from sciatic nerve tissue or Schwann cells cultured *in vitro* were separated by SDS-PAGE and transferred onto PVDF membranes (Millipore, Bedford, MA). After blocking with 5% skim milk in TBS-T at room temperature for 1 hour, the membranes were probed with rabbit anti-rat cox-2(1:500 dilution, CST) or Caspase-3(1:1000 dilution, CST), Cleaved Caspase-3(1:200 dilution, CST)and GAPDH (1:5000 dilution, Sigma) primary antibody at 4 °C overnight and the membranes were washed three times with TBS-T prior to incubation with horseradish peroxidase-conjugated secondary antibody at room temperature for 1 hour. The signals were detected by chemiluminescence using gel imaging system (Bio-Rad Laboratories, Hercules, CA, USA).

### Bio-Plex Pro^TM^ Assays

Bio-Plex Pro™ assays are magnetic bead-based multiplex assays designed to measure multiple cytokine biomarkers. In this study, plasma samples were centrifuged twice after thawing and then IL-1β and IL-6 were measured with the Bio-Plex Pro^TM^ Kit (Bio-Rad Laboratories, Hercules, CA, USA) according to the manufacturer’s instructions. Finally, the results were analyzed using the Bio-Plex 200 system and the Bio-Plex Manager software (Bio-Rad Laboratories).

### Statistical analysis

Data were expressed as mean ± SE. All the grouped data were statistically analyzed with SPSS 13.0. Comparisons between groups were performed by one-way ANOVA or independent t-tests. Comparisons about before and after treatment in same group were performed by repeated one-way ANOVA. The *P* value *<* 0.05 is considered significant.

## Additional Information

**How to cite this article**: Chen, L. *et al*. Thymoquinone Alleviates the Experimental Diabetic Peripheral Neuropathy by Modulation of Inflammation. *Sci. Rep.* **6**, 31656; doi: 10.1038/srep31656 (2016).

## Figures and Tables

**Table 1:** Effect of Thymoquinone (TQ) on the blood glucose of diabetic peripheral neuropathy (DPN) rats.

Time	0 week	6 weeks
Normal group	5.46 ± 0.49	5.96 ± 0.29
T2DM group	5.36 ± 0.59	21.12 ± 0.44
DPN group	22.76 ± 4.17	20.40 ± 5.15
DPN+TQ5 group	23.37 ± 6.45	16.81 ± 3.15
DPN+TQ2 group	22.67 ± 4.76	17.50 ± 3.13
DPN+VitB_12_ group	21.71 ± 4.66	19.51 ± 3.47

Note: Week 0 refers to the 3 days after STZ injection and before TQ administration. Week 6 refers to 6 weeks after STZ injection. T2DM: type 2 diabetes mellitus; TQ2: 2 mg/kg TQ; TQ5: 5 mg/kg TQ; VitB_12_: vitamin B12. Unit of blood glucose: mmol/L.

**Table 2:** Thymoquinone (TQ) reduced inflammatory factor expression in plasma of diabetic peripheral neuropathy (DPN) rats.

Time	IL-6	IL-1β
Normal group	OOR<	OOR<
T2DM group	9.937 + 1.406	376.8 + 57.96
DPN group	10.845 + 1.15	384 + 34.44
DPN+TQ5 group	OOR<**	129.3 + 48.11**
DPN+TQ2 group	OOR<**	305.3 + 57.1
DPN+VitB_12_ group	OOR<**	366.3 + 72.58

Note: 6 weeks after TQ administration, IL-6 and IL-1β expression were detected by Bio-Plex ProTM Assays. OOR<”means much smaller than the minimum detection range. **p < *0.01* versus the DPN group. T2DM: type 2 diabetes mellitus; TQ2: 2 mg/kg TQ; TQ5: 5 mg/kg TQ; VitB_12_: vitamin B_12_. Unit of inflammatory factors: pg/ml.

## References

[b1] Guariguata L. . Global estimates of diabetes prevalence for 2013 and projections for 2035. Diabetes Res Clin Pract 103, 137–149 (2014). 24630390 10.1016/j.diabres.2013.11.002

[b2] Farmer K. L., Li C. & Dobrowsky R. T. Diabetic peripheral neuropathy: should a chaperone accompany our therapeutic approach? Pharmacol Rev 64, 880–900 (2012). 22885705 10.1124/pr.111.005314 PMC3462992

[b3] Tesfaye S. & Selvarajah D. Advances in the epidemiology, pathogenesis and management of diabetic peripheral neuropathy. Diabetes Metab Res Rev 28 Suppl 1, 8–14 (2012). 22271716 10.1002/dmrr.2239

[b4] Ewing D. J., Campbell I. W. & Clarke B. F. Mortality in diabetic autonomic neuropathy. Lancet 1, 601–603 (1976). 55889 10.1016/s0140-6736(76)90413-x

[b5] Vileikyte L. . The development and validation of a neuropathy- and foot ulcer-specific quality of life instrument. Diabetes Care 26, 2549–2555 (2003). 12941717 10.2337/diacare.26.9.2549

[b6] Yasuda H. . Diabetic neuropathy and nerve regeneration. Prog Neurobiol 69, 229–285 (2003). 12757748 10.1016/s0301-0082(03)00034-0

[b7] Chan L., Terashima T., Urabe H., Lin F. & Kojima H. Pathogenesis of diabetic neuropathy: bad to the bone. Ann N Y Acad Sci 1240, 70–76 (2011). 22172042 10.1111/j.1749-6632.2011.06309.x PMC3636709

[b8] Das A. & Mukhopadhyay S. The evil axis of obesity, inflammation and type-2 diabetes. Endocr Metab Immune Disord Drug Targets 11, 23–31 (2011). 21348821 10.2174/187153011794982086

[b9] Agrawal N. K. & Kant S. Targeting inflammation in diabetes: Newer therapeutic options. World J Diabetes 5, 697–710 (2014). 25317247 10.4239/wjd.v5.i5.697 PMC4138593

[b10] Shi X., Chen Y., Nadeem L. & Xu G. Beneficial effect of TNF-alpha inhibition on diabetic peripheral neuropathy. J Neuroinflammation 10, 69 (2013). 23735240 10.1186/1742-2094-10-69 PMC3679954

[b11] Lee K. A., Jin H. Y., Baek H. S. & Park T. S. The protective effects of DA-9801 (Dioscorea extract) on the peripheral nerves in streptozotocin-induced diabetic rats. J Nutr Sci Vitaminol (Tokyo) 59, 437–446 (2013). 24418878 10.3177/jnsv.59.437

[b12] Kawada N. . Towards developing new strategies to reduce the adverse side-effects of nonsteroidal anti-inflammatory drugs. Clin Exp Nephrol 16, 25–29 (2012). 22038259 10.1007/s10157-011-0492-3

[b13] Ragheb A. . The protective effect of thymoquinone, an anti-oxidant and anti-inflammatory agent, against renal injury: a review. Saudi J Kidney Dis Transpl 20, 741–752 (2009). 19736468

[b14] Kaseb A. O. . Androgen receptor and E2F-1 targeted thymoquinone therapy for hormone-refractory prostate cancer. Cancer Res 67, 7782–7788 (2007). 17699783 10.1158/0008-5472.CAN-07-1483

[b15] Salem M. L. Immunomodulatory and therapeutic properties of the Nigella sativa L. seed. Int Immunopharmacol 5, 1749–1770 (2005). 16275613 10.1016/j.intimp.2005.06.008

[b16] Banerjee S. . Antitumor activity of gemcitabine and oxaliplatin is augmented by thymoquinone in pancreatic cancer. Cancer Res 69, 5575–5583 (2009). 19549912 10.1158/0008-5472.CAN-08-4235

[b17] Al W. R. Nigella sativa and thymoquinone suppress cyclooxygenase-2 and oxidative stress in pancreatic tissue of streptozotocin-induced diabetic rats. Pancreas 42, 841–849 (2013). 23429494 10.1097/MPA.0b013e318279ac1c

[b18] Kanter M. Protective effects of thymoquinone on streptozotocin-induced diabetic nephropathy. J Mol Histol 40, 107–115 (2009). 19484499 10.1007/s10735-009-9220-7

[b19] Kanter M. Effects of Nigella sativa and its major constituent, thymoquinone on sciatic nerves in experimental diabetic neuropathy. Neurochem Res 33, 87–96 (2008). 17713854 10.1007/s11064-007-9419-5

[b20] Mehra M., Merchant S., Gupta S. & Potluri R. C. Diabetic peripheral neuropathy: resource utilization and burden of illness. J Med Econ 17, 637–645 (2014). 24888404 10.3111/13696998.2014.928639

[b21] Satoh J., Yagihashi S. & Toyota T. The possible role of tumor necrosis factor-alpha in diabetic polyneuropathy. Exp Diabesity Res 4, 65–71 (2003). 14630568 10.1155/EDR.2003.65 PMC2478597

[b22] Sango K. . High glucose-induced activation of the polyol pathway and changes of gene expression profiles in immortalized adult mouse Schwann cells IMS32. J Neurochem 98, 446–458 (2006). 16805838 10.1111/j.1471-4159.2006.03885.x

[b23] Lehmann H. C. & Hoke A. Schwann cells as a therapeutic target for peripheral neuropathies. CNS Neurol Disord Drug Targets 9, 801–806 (2010). 20874704 10.2174/187152710793237412 PMC4053445

[b24] Wang L. . Phosphodiesterase-5 is a therapeutic target for peripheral neuropathy in diabetic mice. Neuroscience 193, 399–410 (2011). 21820491 10.1016/j.neuroscience.2011.07.039 PMC3391742

[b25] Zhang J., Zhong H. B., Lin Y., Yao W. & Huang J. Y. KLF15 suppresses cell proliferation and extracellular matrix expression in mesangial cells under high glucose. Int J Clin Exp Med 8, 20330–20336 (2015). 26884948 PMC4723793

[b26] Wu J. H. . MiR-18b suppresses high-glucose-induced proliferation in HRECs by targeting IGF-1/IGF1R signaling pathways. Int J Biochem Cell Biol 73, 41–52 (2016). 26851511 10.1016/j.biocel.2016.02.002

[b27] Wang X., Shi L., Han Z. & Liu B. Follistatin-like 3 suppresses cell proliferation and fibronectin expression via p38MAPK pathway in rat mesangial cells cultured under high glucose. Int J Clin Exp Med 8, 15214–15221 (2015). 26629006 PMC4658895

[b28] Gumy L. F., Bampton E. T. & Tolkovsky A. M. Hyperglycaemia inhibits Schwann cell proliferation and migration and restricts regeneration of axons and Schwann cells from adult murine DRG. Mol Cell Neurosci 37, 298–311 (2008). 18024075 10.1016/j.mcn.2007.10.004

[b29] Syroid D. E. . Cell death in the Schwann cell lineage and its regulation by neuregulin. Proc Natl Acad Sci USA 93, 9229–9234 (1996). 8799183 10.1073/pnas.93.17.9229 PMC38624

[b30] Eckersley L. Role of the Schwann cell in diabetic neuropathy. Int Rev Neurobiol 50, 293–321 (2002). 12198814 10.1016/s0074-7742(02)50081-7

[b31] Fukunaga M. . Methylglyoxal induces apoptosis through oxidative stress-mediated activation of p38 mitogen-activated protein kinase in rat Schwann cells. Ann N Y Acad Sci 1043, 151–157 (2005). 16037234 10.1196/annals.1333.019

[b32] Grober U., Kisters K. & Schmidt J. Neuroenhancement with vitamin B12-underestimated neurological significance. Nutrients 5, 5031–5045 (2013). 24352086 10.3390/nu5125031 PMC3875920

[b33] Gan L. . Restorative effect and mechanism of mecobalamin on sciatic nerve crush injury in mice. Neural Regen Res 9, 1979–1984 (2014). 25598780 10.4103/1673-5374.145379 PMC4283280

[b34] Kellogg A. P., Cheng H. T. & Pop-Busui R. Cyclooxygenase-2 pathway as a potential therapeutic target in diabetic peripheral neuropathy. Curr Drug Targets 9, 68–76 (2008). 18220714 10.2174/138945008783431691

[b35] Lee J. S. . EC-SOD induces apoptosis through COX-2 and galectin-7 in the epidermis. J Dermatol Sci 65, 126–133 (2012). 22251572 10.1016/j.jdermsci.2011.12.013

[b36] Kim Y. J., Kim Y. A. & Yokozawa T. Pycnogenol modulates apoptosis by suppressing oxidative stress and inflammation in high glucose-treated renal tubular cells. Food Chem Toxicol 49, 2196–2201 (2011). 21689714 10.1016/j.fct.2011.06.012

[b37] Kellogg A. P. . Protective effects of cyclooxygenase-2 gene inactivation against peripheral nerve dysfunction and intraepidermal nerve fiber loss in experimental diabetes. Diabetes 56, 2997–3005 (2007). 17720896 10.2337/db07-0740

[b38] Cavieres V., Valdes K., Moreno B., Moore-Carrasco R. & Gonzalez D. R. Vascular hypercontractility and endothelial dysfunction before development of atherosclerosis in moderate dyslipidemia: role for nitric oxide and interleukin-6. Am J Cardiovasc Dis 4, 114–122 (2014). 25360389 PMC4212886

[b39] Russell J. W., Sullivan K. A., Windebank A. J., Herrmann D. N. & Feldman E. L. Neurons undergo apoptosis in animal and cell culture models of diabetes. Neurobiol Dis 6, 347–363 (1999). 10527803 10.1006/nbdi.1999.0254

[b40] Russell J. W. . High glucose-induced oxidative stress and mitochondrial dysfunction in neurons. FASEB J 16, 1738–1748 (2002). 12409316 10.1096/fj.01-1027com

[b41] Schmeichel A. M., Schmelzer J. D. & Low P. A. Oxidative injury and apoptosis of dorsal root ganglion neurons in chronic experimental diabetic neuropathy. Diabetes 52, 165–171 (2003). 12502508 10.2337/diabetes.52.1.165

[b42] Chang X. Z. . Identification of the functional role of peroxiredoxin 6 in the progression of breast cancer. Breast Cancer Res 9, R76 (2007). 17980029 10.1186/bcr1789 PMC2246172

